# 3D Printing for Hip Implant Applications: A Review

**DOI:** 10.3390/polym12112682

**Published:** 2020-11-13

**Authors:** Obinna Okolie, Iwona Stachurek, Balasubramanian Kandasubramanian, James Njuguna

**Affiliations:** 1 Centre of Advanced Engineering Materials, School of Engineering, Robert Gordon University, Riverside East, Garthdee Road, Aberdeen AB10 7AQ, UK; o.okolie@rgu.ac.uk; 2 Łukasiewicz Research Network—Krakow Institute of Technology, 73 Zakopianska Street, 30-418 Krakow, Poland; iwona.stachurek@kit.lukasiewicz.gov.pl; 3 Rapid Prototyping Lab, Department of Metallurgical and Materials Engineering, Defence Institute of Advanced Technology (DU), Ministry of Defence, Girinagar, Pune, Maharashtra 411025, India; meetkbs@googlemail.com

**Keywords:** 3D printing, biocompatibility, biomaterials, cell adhesion, hip replacement, tissue regeneration

## Abstract

There is a rising demand for replacement, regeneration of tissues and organ repairs for patients who suffer from diseased/damaged bones or tissues such as hip pains. The hip replacement treatment relies on the implant, which may not always meet the requirements due to mechanical and biocompatibility issues which in turn may aggravate the pain. To surpass these limitations, researchers are investigating the use of scaffolds as another approach for implants. Three-dimensional (3D) printing offers significant potential as an efficient fabrication technique on personalized organs as it is capable of biomimicking the intricate designs found in nature. In this review, the determining factors for hip replacement and the different fabrication techniques such as direct 3D printing, Fused Deposition Modelling (FDM), Selective Laser Sintering (SLS) and stereolithography (SLA) for hip replacement. The study also covers surface modifications of 3D printed implants and provides an overview on 3D tissue regeneration. To appreciate the current conventional hip replacement practices, the conventional metallic and ceramic materials are covered, highlighting their rationale as the material of choice. Next, the challenges, ethics and trends in the implants’ 3D printing are covered and conclusions drawn. The outlook and challenges are also presented here. The knowledge from this review indicates that 3D printing has enormous potential for providing a pathway for a sustainable hip replacement.

## 1. Introduction

Annually, the number of people globally experiencing pains from organ failure or dysfunction from a devastating tissue is on the rise, and this usually affects children and the ageing population. Traumas or illnesses, which include strokes, joint degeneration and heart attacks, can adversely reduce the life quality of the victims and lead to the significant damage of the tissues where new medications are incapable of efficient healing [1].

**Figure 1:**
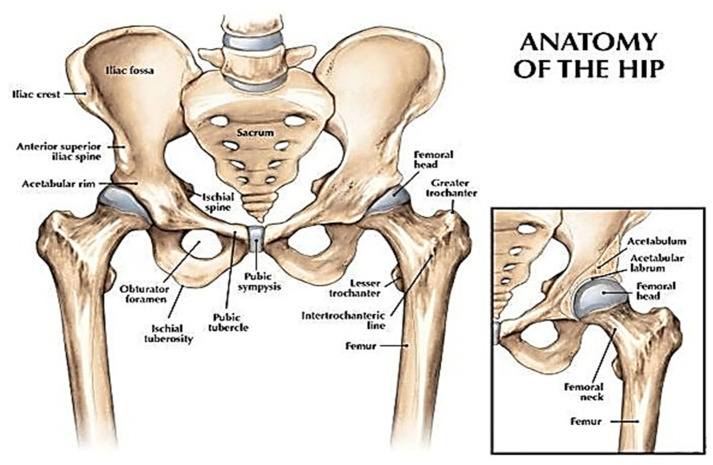
A view of the hip joint [2]. Reprinted with permission from Ref. [2]. Copyright 2007, Lippincott Williams & Wilkins, Inc.

The hip joint comprises two bones which are the femur (thigh bone) and pelvis as illustrated in Figure 1. This joint is the biggest ball and socket synovial joint in the body. This ball is the femur head, which is the rounded edge of the femur while the socket is a curved dip around the lower region of the pelvis (acetabulum). The femur headsets in the pelvis give shape to the hip joint.

Hip pain is common, and it can affect anyone at any age; however, it is most common with the elderly [3]. For instance, in the United States, 76 million people are experiencing some type of pain, and this is a sizeable chunk of the population [1]. It is difficult to pinpoint the specific causes of these pains, but the clues lie in the type of pain and the location of the pain. For most cases of the hip, the pains relate to any problem emanating from the lower back or buttocks region. Meanwhile, the hip problems are relative to the source of the pain in the groin, thigh region and in some cases, the knee because of the hip innervation when the nerve is stimulated [3]. In addition, in the United Kingdom, the National Health Services (NHS) has been supervising the National joint registry for England, Wales, Northern Ireland and Isle of Man (NJR) for all forms of hip surgeries. From the NJR review of 2016, about 101,651 replacements were done that single year which was roughly a 3.5% increase from the previous year [4].

The several conditions which lead to hip pains can be a result of the following: arthritis, injuries, pinched wounds, cancer and other problems such as lifestyle [5]. Arthritis, in particular, is a broadly known condition which refers to pain and inflammation in a joint according to NHS UK and this is mainly due to more than 10 million people currently have this condition which affects people of all ages not excluding children. The cartilage, although flexible, is a firm connective tissue in the joint. The joints are protected when the cartilage absorbs the shock and pressure created when there is movement and stress is applied to them. Cartilage tissue reduction in standard amounts can lead to various forms of arthritis. Osteoarthritis (OA), which is the known form of arthritis commonly caused by normal wear and tear and an injury/infection, will most times aggravate the breakdown of the cartilage tissue.

Non-surgical treatments are considered the first line of action during the treatment of hip pains. Most of the hip replacement implants (90–95%) are durable for at least 10 years; there is a rising public demand for increasing the life span of these implants based on increasing life expectancy amongst the older population with joint degeneration illness [6,7]. As a result, revision hip arthroplasty could be considered when the implant fails due to several reasons such as the aseptic loosening (51.9%), instability (16.9%), infection (5.5%), debilitating pain, periprosthetic fractures or component failure [8]. In addition, there is a limitation on the conventional 2D scans currently, which frequently does not provide well-detailed imagery during implantation. However, this review focuses on the current state of 3D printing technology and the required materials needed to support cell adhesion and growth. Therefore, at this point, a brief review of real-life application of 3D printing needs to be provided.

Hip surgery involves hip replacement and an alternative surgical option for hip arthritis. When the treatments and medications applied do not nip the hip pains to a suitable level, this surgery for reposting or replacement of the hip joint may be the next solution.

About a quarter of the UK population needs a clinical check for musculoskeletal (MSK) conditions to be carried out at least once a year and about 25% or more of all the surgical routines handled by the NHS are mostly based on MSK conditions. There is currently a large variation in the rate of Hip surgeries between the developed countries, and this influences a surgeon’s decision.

In the United Kingdom, the NHS has accepted the value-based healthcare system (VBHC), which has the goal of increasing the obtained values from limited resources that are accessible to the populace [9]. These values in healthcare can be defined as the result that affects the patients and carers that is related to the cost of providing the results. From the study, it was finalized that an approved multidisciplinary team (MDT) consists of experienced service management, orthopaedic nursing staff, physiotherapy and the specialist orthopaedic consultant surgeon. MDT approach permits staff to engage at an enhanced supervisory level, and this facilitates the patients maximizing each result from the treatment. Severe hip deformities present a huge challenge for the MDT that requires the selection of the right form of hip implant, in a bid to attain an error-free replacement with a near-perfect solution to the deformity.

In comparison with the conventional technologies which are solely CT and MRI imaging, a 3D model provides more details for the team and can be also be used by the MDT to simulate the operation. Generally, two main ways 3D printers can be used in hip surgery are either as implants or models. 3D printed implants can be made from suitable materials which are ideal for the reconstruction of huge and unclassifiable acetabular defects. 3D printed models of patients can assist in planning surgery, delivering surgery and teaching surgery [10].

## 2. 3D Printing for Hip Replacement

**Figure 2:**
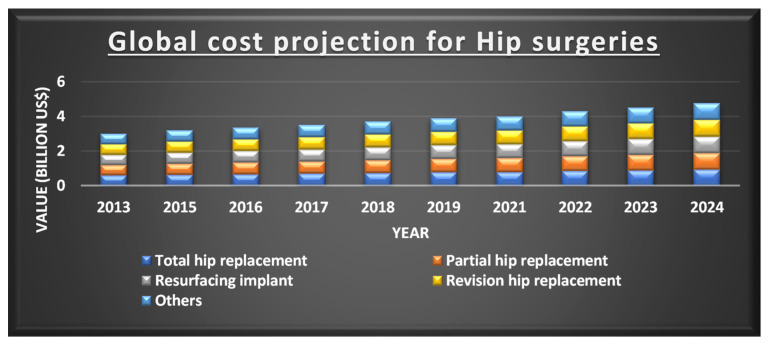
Projection of the cost for different types of hip implants. Reprinted from Ref [11]. Copyright 2019, Polaris Market Research.

There has been a significant improvement in bioimplants in the current decade, where a wide variety of fabrication methods are being applied. In 2018, the global orthopaedic implant is estimated to be at USD 4.747 billion and a compounded annual growth rate (CAGR) of 5.1% is expected from 2019 to 2026 as depicted in Figure 2 [11].

In the human body, the biological functioning is complicated, with the huge differences in biomechanical properties from bone to bone. Such an instance is the elastic modulus of the critical section of denser bones varying from 16–20 GPa; this is a magnitude greater than the trabecular bone. Therefore, it can be understood that certain biomechanical errors are bound to happen between the recently implanted parts and closer bones with similar properties. Furthermore, from a medical perspective, these biomechanical properties may differ greatly from the body to the body. Hence, a need for fabrication techniques that can meet specific geometry for a precise injury/defect is justifiable. Additive manufacturing (AM), also termed rapid prototyping (RP) technology, is a common name for the fabrication technique depending on the idea of surface development. From its emergence in the 1980s, this technique has been garnering research interest in the sector of manufacturing [12,13,14,15]. In contrast to conventional implants, 3D printed implants can be tailored to several forms of diseases [16,17,18]. With the possession of excellent design ability, 3D printed implants can solve certain challenges where it is complicated to insert and repair the different conventional implants together [16,17,18].

The pros of 3D printing include the capability of biomimicking extracellular matrix (ECM) and the ability to fabricate adaptable scaffolds regardless of the shape complexities for the cell distribution to done homogenously. However, the major limitation is the accessibility of suitable biomaterials that possess the stability and intrinsic properties for 3D printing of scaffold. An additional limitation is a time required for scaffold fabrication, and that time increases when the design becomes more complex and accurate [19]. It is worth noting that 3D printers utilize varying powdered mixtures and materials; the size of the structures can easily affect the printability of the scaffold for most materials in 3D printing. For a material to be a viable choice for tissue regeneration, it should be printable with a great degree of reproducibility from 3D printing. These materials should be affordable, effective and malleable to create the morphology required for the designed scaffold. Within the last four decades, various 3D Printing (3DP) techniques have been suggested due to the processing approach. Of importance is the ASTM/ISO 52900:2015 standard [20,21] designated over 50 different 3D techniques which can be grouped as (i) binder jetting, (ii) direct deposition, (iii) material extrusion (FDM), (iv) material jetting/inkjet, (v) powder bed fusion (SLS), (vi) sheet lamination, (vii) stereolithography (SLA, DLP). For this review, the emphasis is on direct 3D techniques which usually utilize several forms in atmospheric conditions such as fluids capable of solidifying, nano fine powdered particles, layered sheets and flexible filaments.

Currently, 3D printing products do not have a formal legal standing that clarifies them both for implantable and non-implantable devices. Using Europe as the base reference, whole 3D-printed products can be classified as customized tools under the regulation (EU) 2017/745 of the European Parliament and of the council of 5 April 2017 [22,23]. It was stated that “any device specifically made in accordance with a written prescription of any person authorized by national law by virtue of that person’s professional qualifications which gives, under that person’s responsibility, specific design characteristics, and is intended for the sole use of a particular patient exclusively to meet their individual conditions and needs”. Varying from mass-produced devices “which need to be adapted to meet the specific requirements of any professional user and devices which are mass-produced by means of industrial manufacturing processes in accordance with the written prescriptions of any authorized person shall not be considered to be custom-made devices” [23]. In fact, manufacturers of customized tools are only assured through a commitment of conformity assessment methods whereby the tools must comply with the performance and safety requirements [24].

The most regularly used 3D printing techniques include direct inkjet printing, bioprinting, powder deposition printing (FDM), laser-assisted printed (SLS) and stereolithography (SLA). Therefore, the study aims to discover methods for novel biomaterial fabrication through 3D printing that can be used in hip implant design, applications and is a biocompatible tissue scaffold. Before the AM technique, 2D slice data are obtained from the designed surface of 3D structures. Required materials are fabricated through the combination of material layers [25]. As opposed to conventional fabrication techniques that take out materials from a whole, AM technique creates 3D materials by continuously adding layers instead. Currently, there is enough evidence of the economical production of these unique implants. The key types of this process are discussed in the sections below.

### 2.1. Direct 3D Printing for Hip Replacement

**Figure 3:**
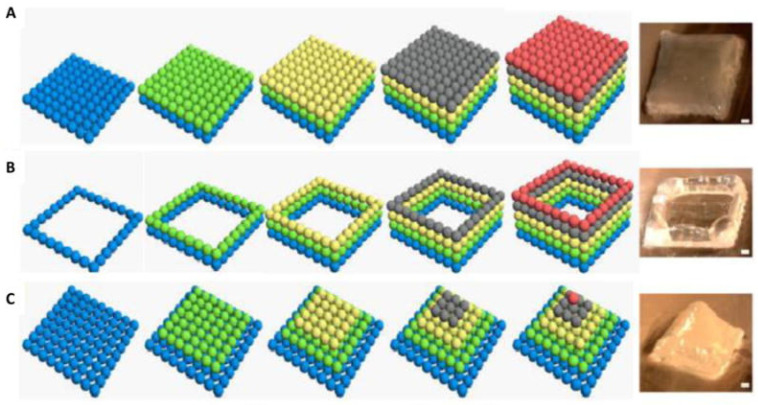
Formation of scaffolds fabricated through inkjet 3D printing of alginate hydrogels through varying geometries: (**A**) square model (**B**) pyramid model and (**C**) scale bar. (1 mm) [1]. Reprinted with permission from Ref. [1]. Copyright 2015, John Wiley, and Sons.

3D printing includes the creation of materials by the accumulation of a successive layer through a computerized technique. The first use of a 3D printer was done in the early 1990s and was centred on the concept behind the conventional inkjet printer, otherwise known as the drop on powder or binder jetting [26]. For a conventional 2D inkjet printer, the nozzle swerves to both sides in increasing the pace where the printed materials are in 2 dimensions of width and length. The 3D printer utilizes similar technology although the side movement goes on a single plane the printer can go top and bottom (90° to both sides). Therefore, the addition of this height makes it 3-dimensional. The recent 3D printer has the same features as that of a 2D inkjet printer, rather than ink, 3D printers utilize a liquid binder solution which undergoes selective deposition that is layered on a powdered bed rather than on paper. In the beginning, the powdered bed which varies based on the type of material applied and is placed on the structured platform and spread evenly by a roller system. The nozzle emits the binder solution into the precise powdered region as directed from CAD. Immediately the combination of the powder and the binder solution occurs, the powder in excess is blown out. The studied platform is then reduced, and an additional powder layer is deposited and spread evenly. Subsequently, this process is continuously repeated, as seen in Figure 3 until the final structure is fabricated.

Millazo et al. [27] developed the palmetto 3D printer and use it to study the viability of creating alginate scaffolds through the addition of CaCl_2_ as a liquid binder. Z corp produced biocomposites consisting of hydroxyapatite (HA) and chitosan via a spectrum of 510 3D with interest to obtain enhanced porous (cylindrical) and dense (solid) scaffolds. The fabrication technique used for these scaffolds was the application of binder solution comprising of varying hydroxyapatite/chitosan composites (20, 25 and 30% chitosan) and lactic acid (40%) and subsequently underwent a post-hardening procedure. The best mechanical properties were noticed when the solid printed scaffolds used 25 wt.% chitosan as the Youngs modulus of 4GPa and compression strength of 16Mpa was demonstrated. This is a popular 3DP technique which is currently in use. It is most times considered an alternative to stereolithography [28].

However, when compared to the extrusion-based technique, it is limited, and this can be attributed to the movement of the printing head to provide a continuous flow of printing solution, which limits the significance in bioprinting. 3D inkjet printing is popular to produce intricate scaffolds in different clinical shapes for biomedical applications, and they are also mobile and portable with synthetic PCL being the most used ink.

Walczak et al. [28] fabricated a microfluidics channel chip for capillary gel electrophoresis. The chip production time was roughly 3 h which makes inkjet printing appealing in contrast to the conventional process. Krivec et al. [29] created a 3D prototype via photopolymer adoption. In addition, silver nanoparticle ink is added for identification of radiofrequency for enhancing wireless radar signalling. Zhang et al. [30] used inkjet 3D printing technique to produce HA-based scaffolds with controlled porosity on a micro/macro size.

Additionally, the mechanical properties (compressive strength) of the 3DP bioceramic scaffolds with various HA nanopowder diameters was achieved. From the Cui et al. [31] study, there is a possibility of damaged cells through the thermal inkjet 3D printing technique. Chinese hamster ovary (CHO) cells were used to analyse cell viability, and the researchers scrutinized the total number of the affected cell membrane and concluded that the thermal inkjet technique is an efficient and trustworthy process for 3DP mammalian cells. A study of 3D thermal inkjet printing technique which was solvent-free was done by Kyobula et al. [32] to create a drug release agent via natural means. The structure of the produced drug was designed in a honeycomb structure that has a regulated cell shape. The study aimed to produce drug release tablet via beeswax in the order of honeycomb structure, which can be taken in a solid dosage form. Inzana et al. [33] evaluated the best mechanical property and biocompatibility of collagen. Calcium phosphate was fabricated by the inkjet 3D printing to optimize phosphoric acid binder solution blending with collagen. This technique improved cell compatibility and potential for enhanced bone regeneration. The research by Xu et al. [34] on an inkjet fabricated 3D cellular structure blended with NT2 cells and fibrin was possible via layer to layer technique with the aim of neural cell sheet production, which has multiple functions in tissue engineering.

The materials currently used in hip implants, such as titanium-based alloys, cobalt-chrome alloys and 316L stainless steel, have a stiffness much greater than bone. When a metal implant is inserted into the femur, most of the physiological load is transferred to the implant, away from the relatively more compliant surrounding bone. The altered load transmission in the implanted femur leads to underloading of the bone compared to its natural state. As a result, bone, living tissue that is sensitive to mechanotransduction, resorbs and loses mass through an adaptive process called bone remodelling. Further the structural stiffness, mainly related to the mass and modulus of the material, requires significant extra engineering structural modifications efforts to reduce the stiffness mismatch between the metal/alloy implants and the adjacent bone. In addition, ceramics and steels are non-porous and the porosity is a necessary feature as it aids in the movement of nutrients, oxygen and cell waste.

The reason for this inefficiency can be attributed to poor hardening technique, and this technique uses a loose concentration of solvent in which the scaffold is immersed in it which subsequently leads to the collapse of the pores [35]. Hence, the creation of 3D printed polymer scaffolds for bio application, albeit a promising future, still requires more research on property optimization.

### 2.2. Fused Deposition Modelling (FDM) for Hip Replacement

**Figure 4:**
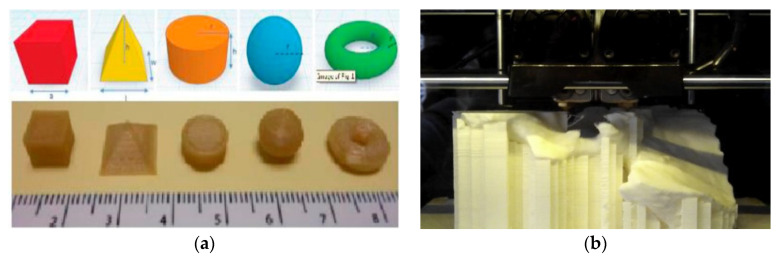
(**a**) 3D representation and images of the 3D geometries printed using the FDM process [38]. (**b**) 3D printing of an acetabulum through Fused Deposition Modelling (FDM) with acrylonitrile butadiene styrene (ABS) polymer [39]. Reprinted with permission from References [38,39]. Copyright 2017, Elsevier, and 2016, Elsevier.

This is a form of 3D printing that involves the use of thermosensitive polymers that undergo heating above its glass transition temperature and a solid medium deposition occurs. FDM is closely similar to SLA, and it was initially used for only 3D polymer structures. The FDM has been developed to produce ceramic, ceramic/polymer composites which is termed a fused deposition of ceramics [36,37]. For this technique, a word thermoplastic polymeric filament is used by unwinding and extruded via a hot nozzle onto a fabrication based. On settling on the platform, the polymer cures. After a series of the layer on layer deposition, the final CAD structure is produced. A schematic representation is presented in Figure 4 below.

The key advantage of FDM is that there is a low possibility of toxicity from organic solvent when they solubilize polymers; for example, dichloromethane used for solubilizing PLGA [40]. They can also be applied on scaffolds without cells or any bioactive molecules which has gradients in x, y and z directions. However, the limitation in the requirements used for determining the right thermoplastic material which hinders its versatility and application range concerning scaffold fabrication with the result being that acrylonitrile butadiene styrene (ABS) is the most common used material, and this makes the basis of this study for feasibility on ease of polymer-based scaffold fabrication. Polymers like polyetherimide (PEI), polyphenyl sulfone (PPSF) and polycarbonate (PC) were used previously [41], and in several studies, polymeric structures have been fabricated through FDM. However, it is only a select few that is biocompatible and suitable for biomedical applications. An example is acrylonitrile butadiene styrene (ABS) which can be sterilized by either ethylene oxide (EtO)/gamma radiation [42]. PCL is the most extensively studied polymer for 3D printed structures. Korpela et al. [43] produced a polylactic acid (PLA) copolymer, poly(l-lactide-co-ε-caprolactone) (PLC) copolymer, PCL bioactive glass, PCL through FDM.

A recent approach to the increment of FDM use is the inclusion of electrospinning. Researchers have been able to utilize a makeshift melt electrospinning device to manufacture hip-based scaffold with a periodontic and bone chambers. For example, medical level PCP/TCP membrane scaffolds behaving as FDM fabricated the bone chamber, and a calcium phosphate coating was applied. In contrast, the periodontic chamber was electrospun via the spinning device. Through compression of a partly fused CaP with the bonded bone chamber into the periodontal chamber, assembling them forms the biphasic scaffold. This scaffold had a pore size varying from 100–400 µm, which is large and makes it possible for cellular diffusion. In vivo tests on the scaffold reckon that there was sufficient integration between the chambers and a great tissue orientation and vascularization levels in both chambers [44]. The inclusion of varying 3D printing methods and new scaffold models can potentially enhance 3D printing technologies with the ability to fabricate better quality scaffolds for tissue engineering. In addition, in another study, Borges et al. [45] evaluated the potential of 3D printing (FDM) of artificial meniscus made of polycarbonate-urethane (PCU) and ultra-high-molecular-weight polyethylene (UHMWPE) i.e. PCU/UHMWPE. It was discovered for the FDM that although PCU/UHMWPE can be fabricated, the introduction of UHMWPE had no effect in terms of reducing wear and friction and this can be attributed to a larger surface roughness regardless of the great level of porosity in the FDM blend. Thus, surface treatment is necessary before application.

### 2.3. Selective Laser Sintering (SLS) for Hip Replacement

**Figure 5:**
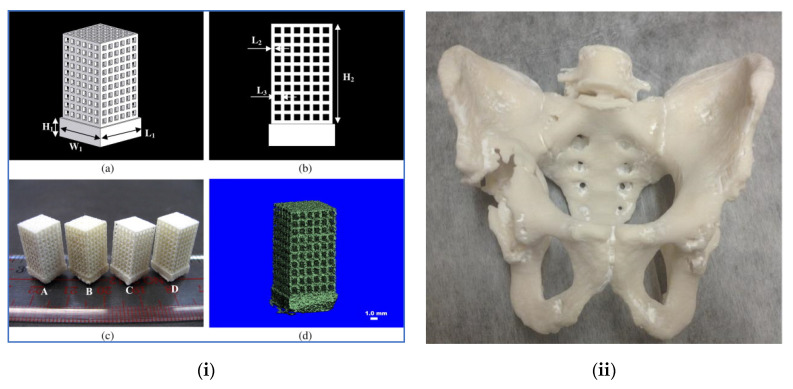
(**i**) (**a**) Schematic diagram of scaffold model PHBV (diametric view), (**b**) scaffold model (Side view), (**c**) scaffold models from Selective Laser Sintering (SLS): (**A**) PHBV, (**B**) CaP/PHBV, (**C**) PLLA, (**D**) CHAp/PLLA. (**d**) MicroCT image of a CaP/PHBV scaffold [47]. (**ii**) A life-size 3D printed model of a patient’s pelvis used in providing visual and tactile aid to the surgeon [8]. Reprinted with Permission from Ref. [8]. Copyright 2017, Hindawi Limited.

This is a form of 3D printing scaffold that uses the powdered medium as the surface for the scaffold printing. This technique involves a laser reproducing the object shape on the powder and subsequently fusing the material in a layer by layer pattern until the object formulation is complete. SLS can be applied in a variety of materials such as ceramics, metals and polymers. The level of precision is solely restrained by powder fineness and laser precision; thus, precise detailing and biomimicry are feasible with this printer as can be seen in Figure 5 [46,47]. Previous scaffolds placement via SLS by biodegradable and biocompatible polymer includes polyvinyl alcohol, polycaprolactone and polyetheretherketone [1].

In a study by Pollack and Shen [48], a biocomposite sludge comprising of silica gel, sodium tripolyphosphate and hydroxyapatite (HA) was used to fabricate scaffolds via SLS. These scaffolds show great mechanical strength of roughly 43.2 MPa, albeit the porosity is poor. However, from in vitro studies, it is possible for the applications of this scaffolds in osteoblast cell growth. The use of the SLS technique is impactful when low porosity and great mechanical strength are of interest in the material. None the less, the disadvantage of this technique is that it requires the powdered material to endure laser heat and also withstand scaffold shrinking during sintering. An additional limitation is when SLS is used in pre- and post-treatment of the powdered material via heat between the crystallization and melting temperatures to decrease the level of shrinkage from the laser to the scaffolds. In addition, the scaffold formation process must endure increased temperatures of roughly 1400 °C based on the material to be used. Current research shows that SLS printers can print scaffolds with either a powdered mixture of PCL [48] or PCL/HA [49] with accuracy. They have a great compression module varying from 52–67 MPa when seeded bone fibroblasts, also noticeable was the bone generation from the in vivo study by µCT data and histological straining [49].

Xia et al. [50] studied novel biomimetic composite scaffold, which is nanoHA/PCL was fabricated via SLS. The results derived indicates that both nanoHA/PCL composite and PCL scaffolds have good biocompatibility. However, nanoHA/PCL scaffolds improved new bone formation efficiency higher than the PCL scaffolds and matched the basic requirements for bone tissue engineering scaffolds. Therefore, nanoHA/PCL has potential in orthopaedic and reconstruction applications. Chung et al. [51] investigated the use of SLS to fabricate PCL composite reinforced with varying volume fractions (10–30%) of tricalcium phosphate (TCP) for tissue engineering scaffolds. The parameter of the composites was optimized by a design of experiments which was applied in each sample. This produced closely fully dense and geometrically handled structured scaffolds with a recommendation on the further study on the scaffolds with suitable load-bearing functionality and adequate porosity. Doyle et al. [52] successfully predicted the elastic properties of SLS printed PCL/β-TCP scaffold through computer modelling.

Sudarmadji et al. [53] investigated the relationships of porosity, and mechanical properties of SLS fabricated polyhedral material for functionally graded scaffolds (FGS). PCL was the chosen material for the study. The compressive tests were carried out to relate mechanical stiffness to porosity. The stiffness range obtained matches that of cancellous bone located in the maxillofacial region. Lohfeld et al. [54] attempted to resolve the issue of poor porosity by testing PA materials, and subsequently PCL scaffold was fabricated via SLS to produce thin stents. From the scan results obtained, PCL had larger pore diameters compared to PA. Ghita et al. [55] studied the fabrication of both virgin and used polyether ketone (PEK) powder samples via high-temperature SLS. The physicochemical results obtained shows that despite polymer degradation of PEK, it has the potential to be combined with modified parameters to produce good quality parts. Porous polylactide structure was produced via SLS as a novel material without using diluents by Melchels et al. [56] and incorporated a resin based on 2-arnal poly (D, L- lactide) macromer. Chua et al. [57] blended PVA and HA, which was fabricated by SLS to understand the feasibility of the developed scaffolds and the result obtained showed that the composite has potential for tissue engineering applications. The use of SLS to fabricate PC and post-treatment of reinforcement was investigated by Shi et al. [58]. The sintered aspect of PC cannot be applied as functional parts due to its poor mechanical feature. Therefore, epoxy resin was used to enhance the mechanical properties. The research concluded that there is potential with PC SLS fabricated materials when the reinforced epoxy resin parameter is optimized. Such a material can be functional when the demand for mechanical properties is not too high. Therefore, SLS has the potential for printing bone tissue engineering.

### 2.4. Stereolithography (SLA) for Hip Replacement

**Figure 6:**
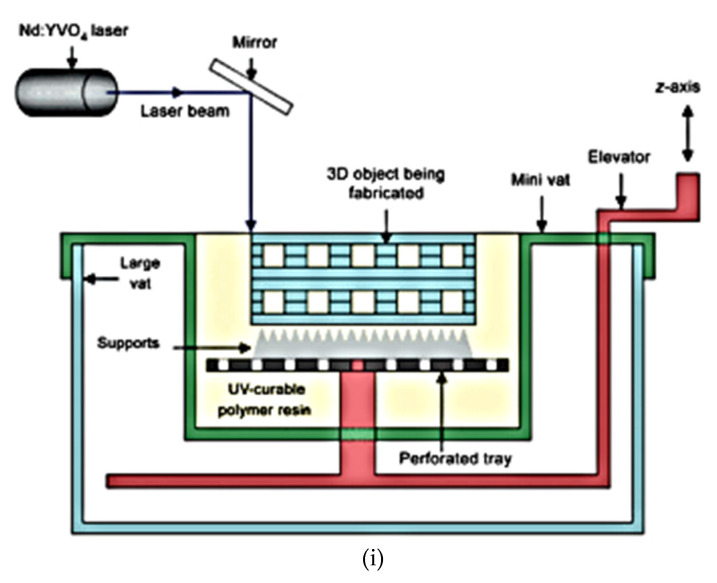

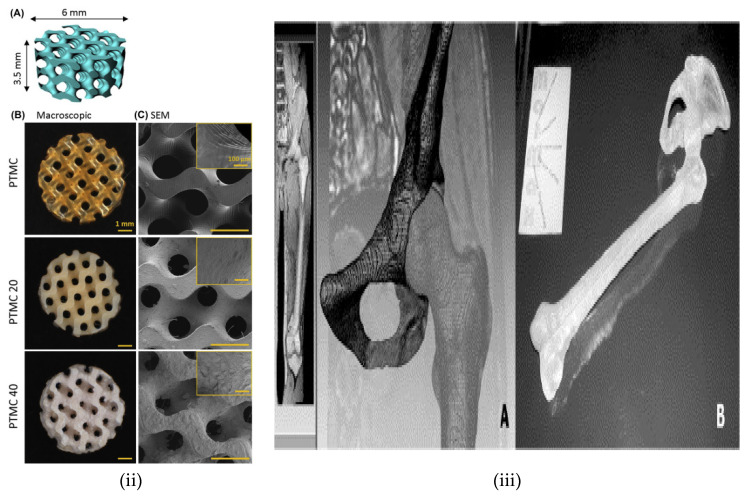
(**i**) Schematic diagram of stereolithography (SLA) printer setup [63]. (**ii**) (**A**) Proposed 3D model, (**B**) macroscopic view of SLA-printed scaffolds containing PTMC only and PTMC with two varying ratios of HA respectively and (**C**) representative SEM images of the SLA-printed scaffolds [64]. (**iii**) A rapid prototype generation starting from MR images, the triangulated mesh is generated. (**A**) The MR image. (**B**) SLA reconstruction of the hip joint [65]. Reprinted with permission from Ref. [65]. Copyright 2005, Elsevier.

This is the first 3D printer which was commercially available, and it is formulated from a liquid polymer source through a light treated chemical reactions. For this technique, a photocurable and photosensitive polymeric material is placed on a surface apparatus and is subsequently opened to a light UV range of 300–400 µm, and also this technique can be utilized when the scaffolds are acellular, complex structures and a single direction of a gradient is required [59,60,61,62]. When initial layers cure, the repetition of this process fabricates the final scaffold design. The schematic diagram of this printer is shown in Figure 6.

PPF is a biodegradable polymer that has been extremely used for tissue engineering due to its ease of fabrication, great mechanical properties and biocompatibility. PPF is traditionally produced through SLA with the use of phosphine oxide as the photoinitiator with diethyl fumarate being the solvent. The process can be optimized by adjusting the factors like laser speed, solution viscosity and power [66]. The benefits of utilizing the 3D printing approaches are the potential control during use and fabricating accurate scaffold geometries with a great resolution that closely mimics the CAD design. Another benefit of the 3D scaffold is localized drug delivery. Through this approach, drugs can be added to the implants, and the drug release can be controlled to increase treatment efficiency and reduce side effects with close tissues. In the study by Lee et al. [66] on embedded bone morphogenetic protein-2 (BMP-2), they aimed to stimulate bone formation through in vivo and in vitro PLGA microsphere on suspension in a PPF/DEF photopolymer. Subsequently, this scaffold was printed through SLA to support BMP-2 for gradual release purposes. The scaffolds boost the regrowth of bone cells by in vivo when compared to conventional gas-forming/leaching method.

The limitation of SLA use is that the material should be photopolymers that can be photoinitiated. The key classification of photoinitiations is radical photopolymerization via hydrogen extraction, cationic photopolymerization (not used in tissue engineering due to by-product emission) and photocleavage study by using SLA 25/40 stereolithographic device for scaffold printing sourced from PPF with the photoinitiator irgacure 8/9 [67]. The scaffold’s pore size was varying from 150 to 800µm with a porosity of 90%. Hence, the viability of PPF material for scaffold fabrication. Cooke et al. [67] also suggested that more in vivo and in vitro tests should be conducted to understand biocompatibility and cytotoxicity. In addition, chitosan is another alternative polymer that is natural and has biocompatibility and biodegradability features that make it ideal for orthopaedic application on bone tissue regeneration. To fabricate chitosan-based scaffold through SLA, irgacure 2959 to boost its photosensitivity with unsaturated monomers and poly (ethylene glycol) diacrylate is being added [68,69]. These scaffolds demonstrated great in vitro cytocompatibility when fibroblast cells are present and enlarged bone tissue regrowth in vitro [69].

Timbleston et al. [70], through combining liquid interface production (CLIP), used stereolithography applied in an approach that supersedes conventional 3D printing techniques based on production time during fabrication. A new layer by layer takes longer to construct and get a fine resolution. With cured phase thickness of roughly 20 µm, the resin height of optical absorption is 100 µm where the fabrication speed was more than 300 µm, with sacrificing an enhanced resolution for speed that supersedes 1000 mm/h was observed when compared to conventional layer by layer 3D printing that fabricates in few millimetres per hour. The CLIP technique functions through a projected UV image via a transparent UV and an opening that is permeable by oxygen below a fluidized resin bath and the cured phase is made between the opening and the continuous high curing section that the reactive fluidized resin is continuously present for curing [70]. There is a continuation of the process where the speed of production limits the resolution of 3D objects. However, the novel application supersedes the regular stereolithography process with respect to its limitation in production time depending on the curing rate of resin and the viscosity instead of the layer by layer printing formation observed in conventional stereolithography [70]. Through SLA, undesirable polymer layers protect the bioglass particles on the surface and can be successfully evaded.

The study concluded the use of the resins and CAD porous structure when precisely fabricated through SLA 3D printing. Good adhesion on the pore-osteoblast to the photocross-linked networks can be noticed. The rate of cell growth on the material was quite similar to that of tissue culture polystyrene and high molecular weight poly (D, L lactide). From the experimental data obtained and interpreted, it shows that PLLA can effectively aid in the improvement of TCP scaffold mechanical properties through SLA. Success in fabricating cell-encapsulated hydrogels from photopolymerizable PEGDA via SLA was achieved by Chan et al. [71]. Furthermore, there was a noticeable viability of multiple cell type deposition and material composition in intricate layers.

There has been a recent innovation of projection stereolithography (PSL) which is used to fabricate custom-based implants. PSLs distinct feature is, rather than the scanned laser in SLA, a digital light-processing chip is utilized to produce photomasks from CAD design to fabricate 2D slices derived from 3D structures, [72] used PSL to process collagen-bond gelation methacrylate (GelMa) hydrogels besides with methacrylamide which enhances photopolymerization of the hydrogel. It was finalized for the different GelMa hydrogel structure that were created with accurate control pore sizes to aid tissue adhesion and growth in the human umbilical vein endothelial cells (HUVECs) which confirms its potency in tissue engineering. SLA is not only limited to soft biomaterials, but also hard materials, e.g., ceramics/polymer composites, and ceramic has been created via SLA. For instance, the study by Elomaa et al. [73] combined a photocross-linkable PCL resin with bioactive glass through the SLA process, and the bioactive glass was evenly distributed via the surface and high porous matrix. The result obtained shows that SLA aids the fabrication of unambiguous composite scaffolds with the bioactive glass being uniformly dispersed on the scaffold surface and readily accessible for rapid ion release and cell bioreactions. The comparison of the different techniques and the reviewed cases of 3D printed scaffolds are listed in Table 1.

### 2.5. Surface Modifications of 3D Printed Implants

Generally, the surface properties of most implants are inadequate in terms of the essential properties required, such as the surface roughness and pores. Therefore, the implant should be modified before its use or further processing such as coating with bioactive materials, both the chemical and physical composition should be modified to derive the needed surface finish. Surface modification technique [87] is vital for the 3D printed implants as it can directly influence the biomaterial–cellular interaction in the surface morphology such as micropores/microgrooves present, roughness, curvature [88]. Interactions of fibroblasts on polycarbonate membrane surfaces with different micropore sizes and hydrophilicity were studied [89]. Surface topography influences several cell properties, e.g., cell adhesion, inflammatory reaction at the implant–tissue interface, cell differentiation, DNA/RNA transcript and protein production [90]. Most of the studies covering this research are based on 2D settings. The technical challenge lies on the difficulty in achieving a uniformly and reliable modification on the surface topography of large porous 3D scaffold. The surpassing of this technical barrier largely helps the holistic biological functionality of 3D products through the enhancement of bioactivity and influencing a rather quick cellular interaction with the body fluids and subsequently leading to cellular differentiation.

The potential of enhancing the surface performance of the scaffold makes electrochemical polishing (EP) very appealing for post surface treatments [91,92]. This was confirmed by the study of Habibzeddah et al. [92] on the hemocompatibility, and biocompatible of EP finished 316L stainless steel. EP also shows good promise for corrosion and wear resistance of biomaterials. Additionally, as earlier stated that cell adhesion and proliferation are necessary for bone tissue replacement, this means that the chemical makeup of the material is sacrosanct, e.g., the hydrophobicity of the materials means that most times there is poor cell adhesion and this should be modified for enhanced bone implants [93].

For 3D porous networks and monolayers, silanization of the surface has proven to enhance cellular bioactivity and adhesion. It also enhances sol-gel and porous bioglass nano porosity [94], reheating [95] and inclusion of nanoHA in the material [96]. Furthermore, the introduction of magnetic nanoparticles to 3DP material has proven to increase proliferation and osteogenetic-based gene behaviour [97]. Most of the study on surface modification has mainly covered cultures and planar surfaces, neglecting the natural topology in vivo, where there are higher cell viability and lower stress in 3D cultures [98,99], with the knowledge that 3DP structures possess adequate porosity and stiffness to enhance bone density gradient. Therefore, the area of future research should focus on combining 3D printing of porous structures with the relevant sol-gel surface modifications for new bone implant surfaces. This makes the composite effective by easing the introduction of additives which includes biominerals/magnetic nanomaterials to boost mineralization and treating properties, respectively.

Herein, this seems feasible due to the low reactivity for most of the biomaterials and techniques presented here, which is the combination of chemical treating, the introduction of additives and 3D printing and should be fully investigated for better synergy. With the rate of advancement from the time of leading to 3D printing being capable of constructing a high degree of structural complex scaffolds. However, these technological advancements have aided in the improved scaffolds, where the macroscopic aspect of the scaffold can be controlled, leading to a high pore interconnected structure. There is still difficulty over the control of surface feature below the microscopic level, which manages the targeted biological reaction. This is a major drawback for materials mostly used for 3D printing and tissue engineering does not influence bioactivity and most bioinert, i.e., polymers [100].

Another procedure dependent on the microscopic structural ability of 4D printed scaffolds is activated through the aid of external structures [101,102,103]. It is also useful in tuning the surface morphological characteristics. The morphological modification at a microscopic level is instigated through massive shrinkage or expansion during 3D printing [104], leading to rearrangement of the surface morphology. However, an emerging strategy is the increase of osteoconductivity of porous scaffolds depending on the layering of inorganic calcium phosphate [105,106,107,108]. This affects the surface make-up of the structure. Several coating methods utilize biomimetic layering of apatite [109], calcium phosphate laser, spray coating of unsintered HA powder, apatite coating through alternate soaking technique [110], HA coupling and calcium phosphate apatite precipitation created at low temperature [111] and have been utilized in developing fabricated surface modification.

For example, Pilipchuk et al. [112] integrated 3D printing and micropatterning of PCL scaffolds (meso and microscale) customized to human ligament-progenitor cells and evaluated its ability to adhere to the bone ligament–cement complexes in vivo. This novel approach was based on the fact that the scaffolds that have combined features of meso, and microscale can align cells in vivo for oral tissue repair and can enhance regenerate reactivity in the bone–ligament cement complexes. SLS was used to manufacture porous scaffolds with precise architecture for possible bioapplication by Ran et al. [113]. To fully evaluate this, porous Ti6Al4V was used as the feed material because of the large pore sizes. The findings discovered provided the basis for custom designs and fabrication of matching porous Ti6Al4V with precise geometry for orthopaedic application.

For bone tissue engineering, the best scaffold design should be able to support bone regeneration process [114]. Material such as silicate bioactive ceramics has been garnering attention in bone tissue regeneration owing to the excellent bioactivity [115,116]. There has been a challenge in efficiently controlling the internal parameters of the porous structure, which includes pore size, distribution, morphology and the outer geometry of the scaffolds. However, most ceramic scaffolds require high-temperature sintering following the printing process, and this often leads to inevitable volume shrinkage, which affects the end product of scaffolds. Yang et al. [114] worked on 3D printed tricalcium silicates (C_3_S) scaffolds which were surface modified through nanostructured topography, being a crystal growth process with C_3_S being the precursor in a mild condition. The results obtained show that the 3D printed C_3_S bone cement scaffolds with modified nano topography surface can be used for bone regeneration applications.

Wang et al. [117] suggested that electroactive particles could have a key part in tissue engineering via controlling cell proliferation and distribution. An investigation on PCL/pristine graphene scaffolds for bone tissue applications can affect the chemical surface modification on the bioactivity level. Results show that the incorporation of pristine graphene had a great impact on cell viability and growth and the surface modification ushers in the improvement of cell response. The means of chemical modification were via 5M NaOH treatment to increase the hydrophilicity. However, there was no significant enhancement of biological performance-based inclusion of pristine graphene. Jackson et al. [98] studied nylon-12 scaffolds by SLS technique and subsequent low-temperature modification of the porous surface with titania and silane sol-gels, magnetic nanoparticle or hydrophobic treatment to understand the versatility of the scaffold. This treated 3D printed scaffold therein can be used in resolving the current challenges around bone grafting issues and bone repairs. In addition, more research should be done to evaluate possible additional material combinations which can impact on other aspects. Although polymers can have a suitable mechanical property, polymers such as PLA also lack bioactivity to aid bone regeneration. Jaidev and Chatterjee [118] developed a surface engineering technique to enhance bioactivity of 3D printed PLA scaffolds. For this process, microporous PLA scaffold was 3D printed with 70% porosity. Chemical conjugation of polyethene into alkali-treated PLA scaffolds and subsequent conjugation to citric acid and coated with HAP. The cellular adhesion and proliferation of HMSCs on the PLA-HAP was roughly 50% greater than that of pure PLA scaffolds which were deduced through the alkaline phosphate activity increase.

**Figure 7:**
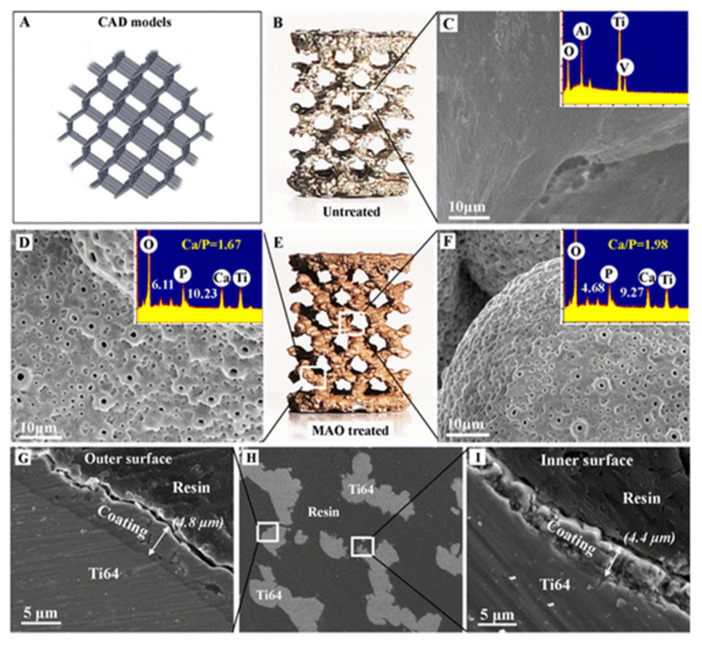
Surface characteristics of the porous Ti64 scaffold before and after micro arc oxidation (MAO) treatment. (**A**) CAD model of multiplanar hexagonal unit cell structures; macroscopical view of the untreated scaffold (**B**). MAO treated scaffold (**C**) and the MAO-treated scaffold at the (**D**) outer surface (**E**); scanning electron microscopic (SEM) images and EDS analysis (inset image) of the untreated scaffold (**F**) central surfaces. Cross-sectional image of the MAO coatings at the (**G**) external and (**I**) internal surface of the scaffold and (**H**) preliminary image shows the positions of the observed struts of the scaffold [121]. Reprinted with permission from Ref. [121]. Copyright 2016, American Chemical Society.

Cold atmospheric plasma (CAP) has also been used by Wang et al. [119] to nanomodify PLA surface. This increased the hydrophilicity and nano roughness, thus leading to better cell adhesion. Visscher et al. [120] successfully produced a dual macro and microporosity PCL scaffold by incorporating 3D printing with salt leaching and suggested its suitability with preventing complications during local drug delivery. Micro arc oxidation (MAO) process has been used by Xiu et al. [121] for 3D printed porous Ti6Al4V (T64) scaffold to fine-tune the scaffold with a uniform layer of microporous TiO_2_ and adequate amounts of amorphous CaP as seen in Figure 7. This proved to be successful with a recommendation of adding extra biologics (e.g., bisphosphonates and strontium) on the coatings and evaluate the osteogenetic effects and bone remodelling in the scaffolds during various physiological conditions.

The hydrophilic characteristics of PCL were enhanced in a research by Lee et al. [122] combining human bone morphogenetic protein-2 (rhBMP-2) and polydopamine (DOPA) chemistry. The result showed a potential application in bone tissue engineering industry. Solvent treatments have also been applied on PCL to enhance the surface with the solvents used being acetone and NaOH. Hydroxyapatite and strontium substituted HA (srHA) was incorporated with PCL by Pierantozzi et al. [123] through FDM to encourage bioactivity reinforcements by mimicking the natural feature of the natural bone. srHAin for PCL/srHA exhibited greater levels of mineralization in comparison to pure PCL and PCL/HA sample scaffolds.

An attempt to enhance bone regeneration through the production of ideal scaffold for enhancing the osteogenic capacity of dental pulp stem cells (DPSCs) was made by Golzar et al. [124]. For the study of 3D printing, β-TCP-based scaffolds reinforced with reduced graphene oxide-magnesium arginine (GRMA)/freeze-dried gelation matrix were fabricated via 3D bioprinter. The study finalized that hybrid β-TCP/0.25 GRMA has the potential to induce cell behaviours which includes viability, proliferation and differentiation. Alksne et al. [125] researched, via in vitro, determining the better reinforcements for PLA between HA and BG (bioglass) with the scaffold fabricated through FDM. The PLA/BG showed better bio interaction and osteoinductive properties concerning pure PLA and PLA/HA scaffold. It was suggested that BG chemical composition is the key difference to the other samples where the BG ions dissociated from BG particles impacted cell growth, bioreactions and osteogenic performance more than HA. Nano HA has also been utilized to improve the mechanical properties of PLA scaffold through printing at different orientations (X-Y plane:0°, 45° and 90°) by FDM [123]. Rheological features at the single-molecule for bone biopolymers like collagen can be taken advantage of when considering materials for hybrids including bioceramics such as barium strontium titanate (BST) and barium titanate to enhance the tensile strength of FDM printed scaffolds [126].

Chronic acid has been successfully used for etching UHMWPE powdered-based scaffolds to create a better design and geometry [127]. Direct 3D printing has been successfully utilized in research on enhancing the mechanical properties, and bone tissue regeneration of potential of Ti6Al4V/HA scaffold provided a highly porous scaffold is achieved [128]. Small amounts of titanium oxide (TiO_2_) have been added to PLA and PCL composites as filler for biomedical application and were printed via FDM 3D printer, which was the study by Nájera et al. [129]. It is noticed via the DSC test that TiO_2_ improved the stability of the composites. There is also a potential for fabricating scaffolds that aid in vascularization. Interconnected foam structure Ti6Al4V has been utilized by Correa et al. [130] to develop a general prototype for a fully living implant structure that can aid in future orthopaedic implants.

## 3. 3D Hip Tissue Regeneration

The bone is the second most-transplanted tissue in the world, with over four million operations using bone grafts or bone replacement materials and these includes hip replacements. The demand for this type of operations is constantly growing. Therefore, the development of bioactive three-dimensional scaffolds (3D) supporting bone regeneration has become an important area of interest in bone tissue engineering (BTE), including the 3D printing method of increasing importance. It should be noted that individual groups of materials, including polymers, ceramics and hydrogels, are not able to reproduce bone properties when used alone fully. However, when groups of materials are used together in 3D composite scaffolds, research shows that you can get beneficial properties and improve bioactivity. Bone is a heterogeneous composite material consisting of hydroxyapatite, type I collagen, lipids, non-collagen protein and water. Therefore, during the production of scaffolds, it is advisable to use a composition of materials to obtain a composite scaffold, and thus potentially enabling greater scaffold bioactivity and structural biomimicry. The bioactivity of the scaffold is also increased by the inclusion of materials that can interact with or bind to living tissues.

On the other hand, increased scaffold bioactivity can lead to better bone cell ingrowth (osteoconduction process), stable anchoring of scaffolds in bone tissue (osseointegration process), stimulation of immature host cells to transform into osteogenic cells (osteoinduction process) and increased vascularization. A perfect 3D scaffold should consist of a biocompatible, biodegradable material with similar mechanical properties to the tissue in which it is to be implanted. The scaffolds are not intended for permanent implants and ideally facilitate host cell deposition of the extracellular matrix (ECM) and replace the scaffold structure over time. Therefore, the 3D architecture of the scaffolding should be very porous with the connected structure to facilitate cell attachment, proliferation and differentiation [131].

Tissue engineering, as it is known currently, is a multidisciplinary field that applies the concept of life sciences and engineering towards the continuous development of natural alternatives. This has rapidly evolved from the area of biomaterial development and entails the process of combining cells, scaffolds and bioactive molecules into functional tissues. The objective of tissue engineering is to gather a functional structure that can repair, preserve and enhance tissue functionality or the whole organ [132]. On the other hand, regenerative medicine/tissue regeneration is a broad field which includes tissue engineering. In addition, it integrates advancement in self-healing, which is a situation where the body uses its system, most times with the aid of foreign biomaterials to reproduce cells and restore tissues and organs.

The goal of tissue regeneration through surgery is to replace damaged/diseased tissues with healthy and performing tissues, tissue regeneration tends to focus on the cure rather than treating complex, often incurable diseases. This has been made possible through tissue engineering, which requires extensive knowledge of the biological process necessary for differentiation and proliferation at the cellular level. This tissue engineering process often starts with a scaffold which is a 3D structure support material required for the suitable differentiation and proliferation of the cells immersed in the scaffold.

The area of tissue engineering and regeneration seeks to address these vital statistics for an improved implant application. This originally involved the transplanting of tissue from one area to another within the same body (Autograft) or from one body to a different body (an allograft) has been effectively used in replacing organs with consistent results [133].

However, there still exist multiple problems with both procedures (autograft and allograft). The autograft technique is expensive and might cause an increased risk of infections, additional injury and is limited due to the unsuitable anatomical replacements from a different body region. While allografts are often not fully accepted by the immune system (immunosuppressant therapies) which is necessary and pose a threat from infection risks and may lead to the possible transfer of illnesses or diseases between the bodies. Most recently, there has been a spike in the study of tissue replacement designs that utilize physical, biological or/and mechanical components to restore functionality [134,135,136,137,138]. Specifically, tissue engineering originally involved the concept of cell isolation from a body, proliferating in vitro and growing them into a biomaterial that is subsequently implanted into the spot of the injury via in vivo. As such, the goal is to fabricate artificial tissues and organs to seek redress for the reduction of risks from the grafting methods (allograft an autograft). Several developed studies supply the needed information regarding how the cells interact with the extracellular matrix hip (ECM) to determine cell behaviour and function [139,140,141,142,143]. ECM in a 3-dimensional structure provides the mechanical support for cells around it. The potential for synthetic biomimicry mechanism development like ECM is an advantage of tissue engineering. A benefit of using 3D printers in replacement is that in certain biomaterials such as thermoplastics, cells can be inserted at the right temperature and precise location to produce a 3D implant that is based on the obtained clinical imaging. Through this process, a strong hip implant is produced that is surgically inserted to heal the bone/tissue deformities, and this implant would biologically degrade with time to leave behind solely natural bone/tissues.

The biomaterials to be used for tissue engineering should have the following features [143]:

(i) Should be porous (to ensure nutrient movement, removal of waste and cell growth), biocompatibility, reproducibility, cell/tissue compatibility, easy preparation and biodegradable.
(ii) Lead to the reduced inflammatory reaction, therefore, decreases the possibility of immune system rejection.
(iii) Advantageous if the biomaterial tissue scaffolds can act as substrates that support cellular fastening, growth and differentiation.
(iv) The cells grow and differentiate, and this scaffold must have the ability to resist the forces put in by the cells else the scaffold disintegrates and causes dismal diffusion of nutrients, waste and oxygen.
(v) The scaffold structure should be mechanically stable to be capable of maintaining load-bearing and varying body movements in daily activity on the joint.

Hip replacement has witnessed a rapid advancement over the past decades, and the specific techniques for surgery have evolved. With the continuous advancement of hip replacement, the biological knowledge of orthopaedic tissues continues to advance. Likewise, the demand for biological solutions for pre and early defective hip remains a challenge for surgical hip treatment [144]. Furthermore, within the hip, there is a rich presence of vascular tissues, and this results in complexities during hip replacement surgery as adjacent vessels could be damaged during operation, an efficient preoperative planning procedure would significantly prevent this [145]. Other vital orthopaedic tissues include the articular cartilage, labral fibrocartilage and Ligamentum Teres [144].

Tissue preservation or minimal invasive total hip replacement (THR) is currently becoming a priority with the focus being to reduce hospital stay, improve rehabilitation and faster patient recovery [146]. The regenerative process replaces and renews the stem cells to facilitate the preservation, restoration and re-establishment of optimum functionality for tissues and organs. At the early stages of some hip defects, simple injections of stem cells to the hip can position it to regenerate and heal the damaged tissue and bone cell lines [147]. The progress noticed in drug testing and regenerative therapy can significantly benefit from bioengineered human tissues developed via several cell types with precise 3D structure. However, there is a limitation with the production of human tissues which are greater than the millimetre size, and this is due to a lack of techniques for fabricating tissues with embedded life-supporting vascular networks [148].

In brief, the important role in hard tissue engineering and regeneration is played by intelligent biomaterials and structures that exert an instructive/inductive or triggering/stimulating effect on cells and tissues by engineering the material’s sensitivity to internal or external stimuli (e.g., pH, temperature, ion strength and magnetism, favouring the repair and regeneration of damaged tissue). The second group is biomaterials that exhibit intelligently tailored individual properties and controlled functions to actively participate in tissue regeneration in a valuable way. There are some excellent works in the literature such as by Wang et al. [134], Ooi et al. [135], Yorukoglu et al. [47], among others where this subject has been dealt with in depth and readers are referred to these works for further reading.

## 4. Challenges, Ethics and Trends in 3D Printing of Implants

Although there is an improvement of 3D printing that has led to the scaffold fabrication in nanoscales which has varying tissue applications, this process of adopting 3D printing for clinical applications is lagging. Recent obstacles to the development are in the aspect of biological, cost, engineering and administration/safety [138]. For bioactivity, it is required that the necessary activities of the cells, which include cell migration, oxygen diffusion and vascularization grades, should be considered due to the below-par performance. For engineering, the reproducibility and producibility of the scaffolds must be required to establish a homogenous and consistent utilizations.

Application of 3D printing has seen a rapid expansion in terms of the biomedical devices. In addition, with 3D being a multidisciplinary area of biology, material science, there are still challenges that should be checked in the future [139,140,141,142]. Within the last 20 years, there has been an evolution of porous ceramics modification [139,140,141]. However, there are still issues on reproducibility for these porous ceramics being non-existent. A large quantity of metallic implants is now being fabricated through 3D printing with either laser or E-beam-based process, which is dependent on past designs. In the aspect of commercial outlines, porous implants for loading bearing application is still poor.

As earlier stated, procedures for THR vary following different countries and administration put in place. The issue of bone replacement and preservation being necessary for a good quality of life is not just based on current trends. The concept of proximal load transfer results in the continuous attempt to enhance the joint stability and rapid rehabilitation phase. Commercial manufacturing of these implants promises a faster track or minimal surgery as the major goal. This can only be affirmative when research and simplicity converge [141]. Otherwise what matters is not the outcome after a few days but rather a possible permanent solution. Continuous development is a must and should be done in a critical, honest and continuous manner. Implanting scaffolds in patients varies with developing and marketing the products [143].

Furthermore, 3D models developed by Tserovski et al. [144] indicated an improvement in diagnostic accuracy and contributed to the predetermination of the implant and the implant size. In addition, the reconstruction of deformed complex acetabular was planned and executed efficiently with an acceptable surgical outcome. This level of accuracy of the model led to a safe preoperative planning and a precise joint reconstruction. However, the 3D models showed some drawbacks, which are the technically demanding process involved, which needs advanced computer skills and extra training. More so, it is a time demanding process that can take hours (~12) to complete each model. Although these giant strides have been made in recent decades, the following should provide a brief idea on where to apply major focus.

As 3D printing is increasingly utilized in several orthopaedic applications, this technology must be reliable and consistent in delivering high-quality implants which are needed. Therefore, the quality assurance of these implants is crucial. There are still various challenges facing the implementation of quality control measures and they are discussed in this section. To ensure 3D printed implants meet the required standard, the quality of the material used is an important factor. However, most manufacturers are experiencing difficulties with material qualifications. A major factor that is making it complex is maintaining purity of powdered material used in additive manufacturing. It is very easy for powders to get contaminated. Another challenge is when leftover powders during printing are reused. Although reusing can assist in waste reduction, it is worth noting that repeated reuse of the powders like this can alter the particle composition because there is a likely absorption of moisture, nitrogen and oxygen, there is also a possibility of oxidation. Therefore, testing procedures for 3D printing is necessary for ensuring that there is no contamination. CT scans have been suggested as a precise method of detecting contaminations in powders as it provides a useful and robust imaging processing methods which generate detailed reports on material porosity, pore and particle morphology and also shape and size distribution of particles. This is useful in validating raw materials.

Another challenge is the broad range of variables from the 3D printing technique that can influence the structure and form of the implant. These variables cover the total 3D printing workflow, which spans from design to the fabricating and post-processing. Some of these variables are the design of the support structure and the number of times for powder reuse. Currently, manufacturers use a trial and error approach to handle the various variables to obtain a technique that can reproduce implants. However, these may lead to the manufacturing of the end product multiple times and requires extensive testing of the implant. A closed-loop quality control system has been suggested as the solution for this anomaly, for this the integration of three elements is required for a more reliable, faster and sustainable quality assurance for a 3D printed implant and the elements are planning the structure through simulation, in-process monitoring of the build process and feedback control that spots deviations of parameters during printing and automatically repairs the structure to balance them. Finally, human error is another challenge which poses the biggest risk in ensuring 3D printed implant meets the necessary standard required. This can be attributed to 3D printing still needing more human intervention than is expected, and this comprises the design, support removal to post-processing and the manual inspection requirements to converge at each stage. With the level of human interventions, it unavoidably increases the risk of compromising the end product. Therefore, quality management is essential in this area [145,146].

3D printing is still a relatively emerging process, and a lot of certifications and standards are still being developed. For the future, the industry needs to have to develop protocols, reference data and testing methods to reduce the time and cost for the qualification of 3D printed implants and processes. As of now, certain standards have been developed for classifying and qualifying the machines and processing techniques of 3D printing such as ASTM/ISO 52900:2015 and ASTM F3303, but they do not cover bioimplant applications. The next phase of this development is quality assurance, support of the implant qualification and post-processing of the printed implants. In addition, the National Institute of Standards and Technology (NIST) is working on developing the quality assurance standards of the additive manufactured products. The current qualification project of NIST is on the methods, metrology and measurements needed for developing a significant understanding of the mechanical performance, qualify 3D printed components and create an efficient post-process measurement [145].

3D printing for biomedical applications involves a lot of ethical issues which surpasses freedom to fabricate any biological device with any biomaterial source. However, the therapeutic potential of those biodevices are enormous and, given the novelty of this technology which involves invasive body alterations, 3D printing treatments need ethical challenge management especially with clinical trial stages because there is need to prove its efficiency before application for treatment purposes as the patient, which the fabricated product was used on, is supposed to serve as a sort of guinea pig [147,148]. Therefore, it would be morally reasonable for patient and society safety to be the priority. As a result, this technology should be fully evaluated, articulated and communicated about nature, objectives and risks involved by the manufacturers and clinical professionals.

Although most countries have administrations that cover these issues such as Merger procedure regulation (EC) 139/2004 (European Union), House of Commons Science and Technology Committee Regulation of medical implants in the EU and UK, Food and Drugs Administration (USA), National association for food, drug and administration control (Nigeria), they cover a general application. More fit for purpose regulations should be provided. This might be difficult to execute continuously. A viable framework is crucial to fill in the gap currently. The framework should answer the following parameters:

- Consideration of limits to bioprinting in medicine.
- Key risks of major harm on the human body because of 3D printing.
- Clinical trial process for bioprinting samples.
- The extent of replicability, irreversibility damage and loss of treatment opportunity during surgery.
- Current ethical laws are guarding 3D bioprinting for bio application.
- The clinically proven advancement of 3D printing over conventional treatments with significant success rates.
- The assurance of 3D printing efficiency in the human body risk to benefit ratio.

These regulations are effective in mitigating some of the possible negative impacts because of implantations. There remains a need for further investigation of the degree of uncertainty and unknown risk linked to 3D bioprinting.

## 5. Conclusions

3D printing technology of scaffolds is a research area rapidly experiencing growth. The aim of any 3D printed scaffolds is for a scaffold fabrication that closely mimics the hip location which is the ECM properties, mechanical strength (load bearing and stress-shielding), adequate pore size for the movement of nutrients and cell growth. With the discovery of new materials, bioinks and efficient printing techniques, there will be an advancement in terms of efficiency and sophistication for hip bioimplants. Scaffold product requires various factors such as biological and mechanical engineering, and this entails the integration of several processes of hip mechanics evaluation, selection of material, fabrication process, surface treatment and study of finished products. Therefore, this research needs to follow this order similar to, as stated above. Noticeably, composites and hybrid materials seem to spearhead the future production of 3D printed scaffolds in tissue engineering utilizations. These materials, as explained earlier, demonstrate potentials in controlling the limitations of any printing technique and the disadvantages of any biomaterials.

As recent development shows potential for enhancement in the current approach, the emphasis of 3D printing in current medicine lies on tissue replacement/regeneration in vivo and probably in vitro. For the successful use of these scaffolds, they should be thoroughly evaluated both in performance and quality. Eventually, it is expected that these scaffolds for tissue engineering will play a larger role in healing hip pains from damaged/diseased tissues and create a pre-ailment or improved living quality. In conclusion, although there is a hindrance now, 3D printing technology has advanced at a fast pace where it can be applied in hip replacements. Nonetheless, for 3D printing improvement for THR, the following is necessary; processing scalability, control and material performance should be key branches to further research. The writer encourages every reader to partake in this research and contribute to a progressive outcome.

## Figures and Tables

**Table 1:** 3D printing applications and the comparison of various techniques used in scaffold printing for tissue engineering.

3D Printing Technique	Composition	Application	Advantages	Disadvantage	References
Direct 3D printing	Alginate/CaCl_2_; HA; Collagen/CaP; Ti alloys	Tissue engineering Bone tissue engineering	Support is not required for intricate structures Multiple uses of suitable materials.	Poor mechanical strength in comparison to SLS. It takes longer time to post-process. Capable of being toxic due to incomplete removal of the binders	[1,30,33,35,74]
Bioplotting	PCL; Nanocellulose-Alginate; Glass-ceramic; Nano CaP/ (PLLA); PLGA, TCP/COL	Tissue engineering Hard tissue engineering	Useful for soft tissues Print of functional cells	Support is required for intricate structure printing. The size of the nozzle limits it.	[1,74,75,76,77,78,79]
FDM	PLA copolymer, PLC copolymer, bioactive glass; PLGA; PU; PCL. HA/PCL, TCP/ PCL; PLGA and PCL; PEEK and ABS; PCL; PCU/UHMWPE	Cartilage tissue engineering Bone tissue engineering Tissue engineering	Relatively low costs for the material and printer Reduced toxicity compared to direct 3D printing	Poor resolution Support is required for complex structure printing Post-treatment is necessary Non-biodegradable materials are used Material limitations that rely on thermoplastics	[1,40,43,45,80,81,82,83,84]
SLS	NanoHA/PCL; PCL/TCP and β-TCP; PCL; PA; PLA; PEK; PVA/HA; PC; Ti alloys; cobalt-chromium; stainless steel; Ni-Ti alloy	Tissue engineering Soft tissue engineering Hard tissue engineering	Enhanced resolution Fabrication of scaffolds with great mechanical strength. The powder gives support to complicated structures.	Higher temperatures usually needed about 1400 °C. The material should be shrinkable and heat resistance. Time and cost extensive via processing/post-processing.	[1,43,50,52,53,54,55,56,57,58,74,85]
SLA	PPF; PEG; PEGDA; GelMa hydrogel; PCL resin; PCL	Bone tissue engineering Drug delivery Tissue engineering Soft tissue engineering	Great resolution Fabrication speed and Surface finish is smooth.	Support is required for intricate objects Cost extensive Biomaterial should be photo polymeric	[1,43,67,69,72,73,86]
